# Small-Sized Co-Polymers for Targeted Delivery of Multiple Imaging and Therapeutic Agents

**DOI:** 10.3390/nano11112996

**Published:** 2021-11-08

**Authors:** Julia Y. Ljubimova, Arshia Ramesh, Liron L. Israel, Eggehard Holler

**Affiliations:** 1 Terasaki Institute for Biomedical Innovation (TIBI), 1018 Westwood Blvd, Los Angeles, CA 90024, USA; ljubimova1@gmail.com; 2 University of California, Los Angeles, CA 90024, USA; arshia6254@g.ucla.edu; 3 Department of Neurosurgery, Cedars-Sinai Medical Center, Los Angeles, CA 90048, USA; liron.israel@cshs.org

**Keywords:** poly(β-l-malic acid) tri-leucine copolymer, multi-ligand carrier, mini-nano carrier, biological barriers, blood–brain barrier (BBB), brain tumors, Alzheimer’s disease

## Abstract

Research has increasingly focused on the delivery of high, often excessive amounts of drugs, neglecting negative aspects of the carrier’s physical preconditions and biocompatibility. Among them, little attention has been paid to “small but beautiful” design of vehicle and multiple cargo to achieve effortless targeted delivery into deep tissue. The design of small biopolymers for deep tissue targeted delivery of multiple imaging agents and therapeutics (mini-nano carriers) emphasizes linear flexible polymer platforms with a hydrodynamic diameter of 4 nm to 10 nm, geometrically favoring dynamic juxtaposition of ligands to host receptors, and economic drug content. Platforms of biodegradable, non-toxic poly(β-l-malic acid) of this size carrying multiple chemically bound, optionally nature-derived or synthetic affinity peptides and drugs for a variety of purposes are described in this review with specific examples. The size, shape, and multiple attachments to membrane sites accelerate vascular escape and fast blood clearance, as well as the increase in medical treatment and contrasts for tissue imaging. High affinity antibodies routinely considered for targeting, such as the brain through the blood–brain barrier (BBB), are replaced by moderate affinity binding peptides (vectors), which penetrate at high influxes not achievable by antibodies.

## 1. Introduction

**Figure 1:**
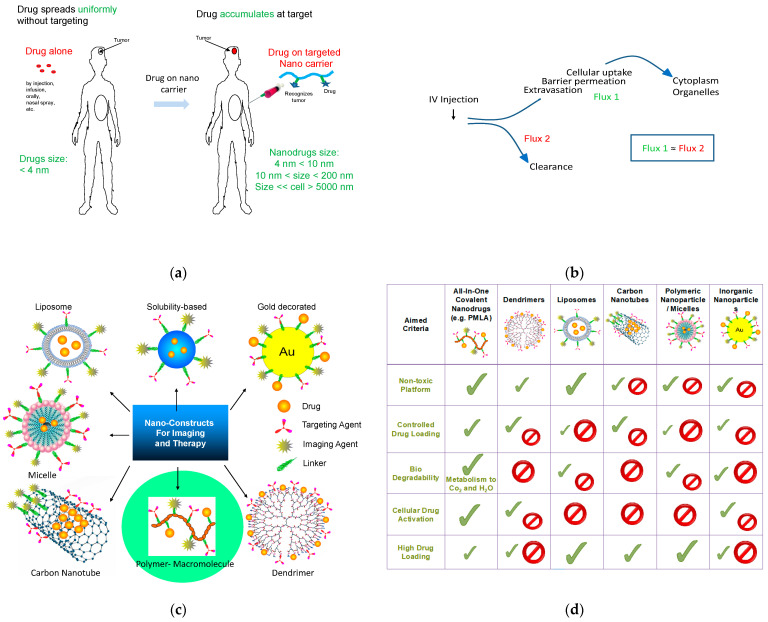
Nanodrugs: function, clearance and extravasation, architectures, and biocompatibility. (**a**) Targeting and accumulation, (**b**) balanced tissue penetration and body clearance, (**c**) drug encapsulation and open covalent structures as polymer-drug conjugates, and (**d**) varying tendency for recipient toxicity.

The invention of nanometer-scale drug delivery was motivated by the possibility of accumulating high drug concentrations at diseased sites through targeted delivery. Optimally, this approach should facilitate the destruction of pathological cells, tissues or organs while leaving healthy regions of the body unaffected [1] (Figure 1a). Given that the body is composed of multiple compartments, a targeted nano drug injected into the bloodstream could find its site by specific guidance across bio borders acting as gated barriers before reaching its ultimate destination for pharmaceutical activity. In order to compete against undesirable clearance, the ligand–carrier conjugate must be competitive from the moment of injection to exiting from the vasculature in a flux (mass delivered per unit of time) comparable to systemic clearance (Figure 1b).

A nanoparticle is a physical entity that contains one or several components (platform, drug, targeting device, imaging agent, and so on), which alternatively function as a multi ligand drug (nanodrug) or imaging agent (nanoimaging agent), among others, having a (hydrodynamic) diameter of 5–100 nm. The lower and upper size limits are not sharply defined. The lower could include diameters of 3–7 nm, the highest being 200 nm or even 1000 nm. The devices with the smallest diameters were termed “mini-nano devices”. The size, shape (e.g., sphere, rod), composition (e.g., helix-coil interchangeable copolymer, shell, solid body), and “stickiness” of a delivery platform can determine its efficiency in moving through receptor gated bio barriers. Mass transporters with long half-lives have been designed to increase delivery, especially when flux (mass delivered per unit time) across a bio barrier is low. Nano capsules such as liposomes, micelles, and sponge-like solid nanoparticles are frequently used as agent carriers [2,3,4] (Figure 1c).

To achieve long-lived transporters, the nanoparticle chemistry, stability, shape, and penetration were optimized, offering delivery of large payloads and prolonged serum pharmacokinetics (Figure 1d). Potentially toxic chemistry and drug leakage were reduced by chemical crosslinking. Scavenging by the reticuloendothelial [1] system has been minimized by attachment of polyethylene glycol (PEG) to create “stealth” particles. For treatment purposes, the highest possible drug loading was often chosen over pharmacologically adequate doses [5]. However, high drug loads carry the risk of side effects (especially in the absence of targeting), excess drug leakage into healthy tissues, cytotoxicity of the carrier or its degradation products, and development of storage disease owing to aggregation and the lack of degradability [6,7,8,9,10,11,12].

### 1.1. Mini-Nanodrugs

Mini-nanodrugs are border-sized linear structured molecules, which are best described as having properties of small chemo therapeutics, yet are loaded at multiple sites for achieving multipronged treatments (Table 1).

Mini-nanodrugs of small-sized platforms are supposed to have low risks of side effects [13]. In comparison with regular-sized nano drugs (>20 nm hydrodynamic diameter), they offer a facile movement through tissue. It is important that the platform can offer a desired number of groups for covalent linkage of ligands, thereby minimizing leakage and toxicity [6,7,14]. Here, we choose poly (β-l-malic acid) (PMLA) as the multi drug delivery platform, but other polymers with multiple ligand binding and high aspect ratios, structural dynamics, and reactivity function well.

### 1.2. Criteria Ruling the Design of Mini-Nano Carriers

#### 1.2.1. General Structure, Function, and Desired Effects

Mini-nano carriers are designed with properties that are shared with low molecule pharmaceutics and full-sized, usually encapsulating nanoparticles. Several of these features are summarized in Table 1.

#### 1.2.2. Example of Mini-Nano Carriers, Composition, and Outstanding Properties

**Figure 2:**
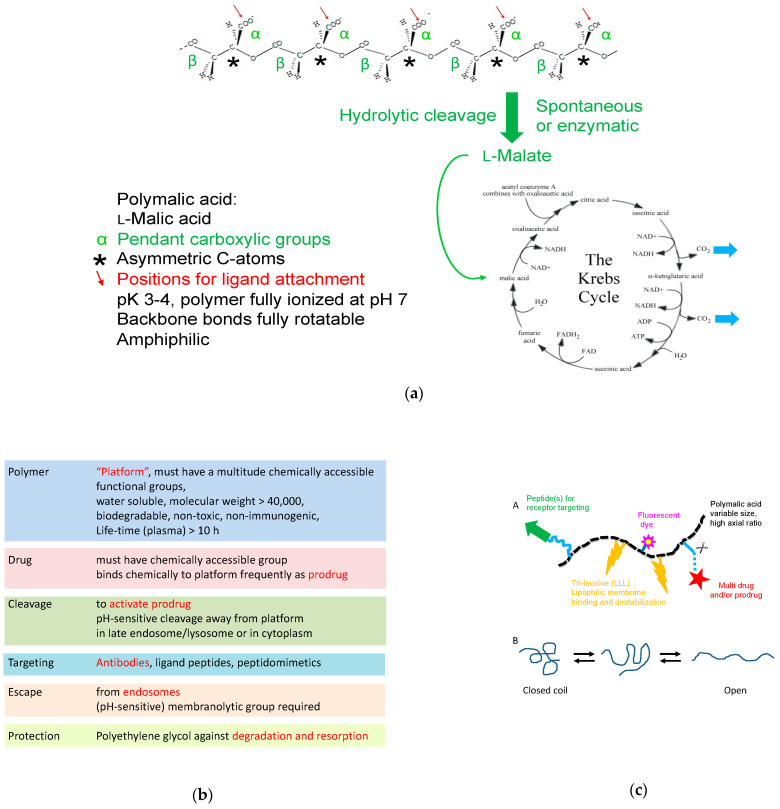
Open-structured border-sized linear nanodrug. (**a**) Polymer nano-platform, biodegradable to l-malic acid, a substrate of the Krebs-cycle. (**b**) Typical functions of the polymeric nano drug. (**c**) Nano drug, schematic composition by ligands (**A**) and dynamic structure (**B**).

To build an efficient mini-nano drug, we chose the macromolecular mini-nano carrier platform PMLA, with a plethora of pendant carboxylic groups for ligand attachment [14,15,16,17,18,19,20,21,22,23,24] (Figure 2). The polyester, which is biologically synthesized by poly-condensation of l-malic acid, can account for a molecular of mass of 30,000–300,000 g/mol, corresponding to 258–2590 carboxyl groups per molecule polymer [25]. Biosynthesis is coupled to the fermentation of glucose by *Myxomycetes* [26,27], and synthetically accomplished by ring-opening polymerization [28,29,30]. Low molecular mass PMLA < 10,000 g/mol is produced in high rates by *Aureobasidium* fungi [31]; however, this source has not been used for the production of mini-nano carriers.

**Figure 3:**
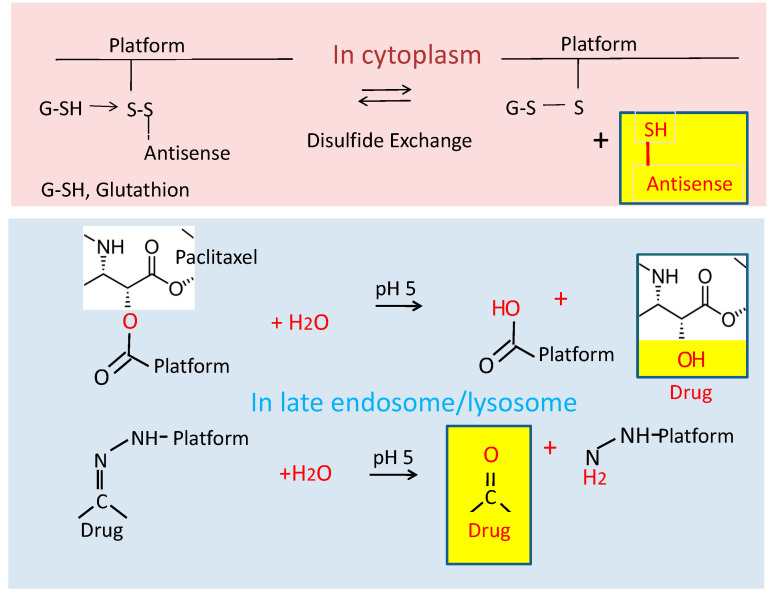
In vivo reversion of synthesized nano-prodrugs into active nano drugs. Examples are disulfides (upper ester) or ester and hydrazone linkages (lower).

The carboxylates are chemically activated by *N*,*N*′-dicyclohexyl carbodiimide (DCC) chemistry in the form of the *N*-hydroxy succinimide (NHS) ester, serving the conjugation of prodrugs and targeting molecules [25]. Bifunctional linkers are commercially available to synthesize multiple bioalkyl derivatives of pharmaceutics (amides, ester, disulfides, and thioethers of oligo nucleotides, peptides, and proteins), achieving nanoconjugates of >1,000,000 g/mol with hydrodynamic diameters of 20–30 nm [14]. Owing to their polymeric platform (>100,000 g/mol), these high molecular nanoconjugates are similar to mini-nano drugs, except for their high molar mass when conjugated to several molecules of antibodies or other macromolecules. In contrast, mini-nanodrugs contain platforms to 40,000–60,000 g/mol. If chosen solely for their high affinity receptor binding, antibodies can be replaced by low molecular weight affinity peptides [17]. In cases concerning biological activities, antibodies should not be replaced unless such activities can be synthetically added. Mini-nanodrugs are often prodrugs. The active drugs can be released by hydrolytic or disulfide cleavage shown in Figure 3.

Examples of mini-nano devices are shown in Table 2, including mini-nano carriers (MNCs), mini-nano imaging (MNIAs) agents, mini-nano drugs (MNDs), and their attached peptide vectors. Synthesis, structure, size, and zeta-potential characterize these linear macromolecules as having border-line hydrodynamic diameter < 10 nm, zeta-potential of −2.2 to −16.5 mV, and molecular mass of (11.4–207) × 10^3^ g/mol. The examples of 50 kDa PMLA platform contain an average of 431 l-malyl-residues, 172 molecules of LLL, 8.6 molecules of vectors (peptides), and 4.3 molecules of rhodamine as the fluorescent reporter [17]. 

Peptides not only imply reduced nano drug size, but also increased conjugate robustness during synthesis, storage, and shipping, as well as the facilitation of medical handling, reduction costs of synthesis, and diminishing of the risk of immune recognition (reduction of antigen determinants) [48,49]. Encapsulating platforms such as spontaneously formed micelles and liposomes or water-insoluble precipitation-fabricated solid carriers are not applicable for highly soluble conjugates. Because of covalent binding, mini-nano drugs do not bear a risk of drug leakage or spontaneous platform dissolution [7]. However, after arrival at the destination site, the delivered drug must be accessible and react with host biomolecules. This conversion from prodrug to drug does not only attest to the precision of site-specificity delivery, but also the imbedding in the nanoconjugate structure can lower the risk of immune recognition [50,51].

Thanks to their structural flexibility, linear structured nano drugs can move deeply into tissue by dynamically adaptation in size and shape. PMLA is a good example for high flexibility, because of its all-backbone single bonds, which allow open and closed structures in dynamic equilibration (Figure 2c(B)). The open forms can readily interact through attached ligands with biomolecules in their microenvironment, as demonstrated by sec-HPLC analysis [15] or assayed by way of covalent fixation [16] Figure 2c(B). Small particle size and low aspect ratios (elongated shapes) support movement through physically porous bio barrier and into deep tissue [17,18].

#### 1.2.3. Permeation through Barriers by Spontaneous Diffusion or Receptor-Gated Access

##### Extravasation

**Figure 4:**
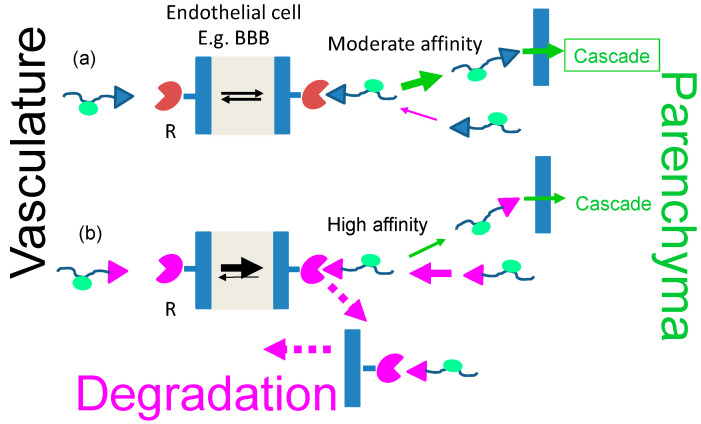
Affinity of the receptor complex with the vector-nano drug cargo determines the success of disease treatment (**a**). Example of moderate binding affinity favoring dissociation from the transcytosis receptor after BBB permeation. After the dissociation, the free vector-nanocarrier-cargo moves away in the cascade downstream towards the diseased target (route **a**). Receptor blocking due to stickiness may favor retrograde permeation and eventually lysosomal degradation (route **b**).

**Figure 5:**
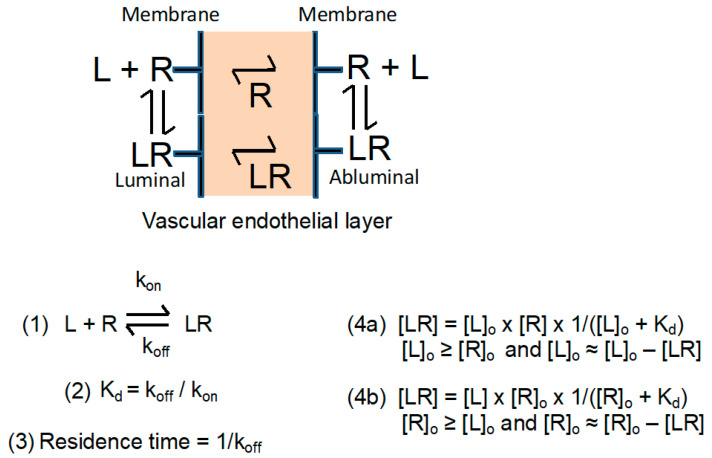
Simplified assumption for ligand–receptor complex (LR or RL) formation and dissociation on the luminal and abluminal sides at vascular endothelial barriers (the first barrier after Extravasation). Symbols refer to k_on_, rate constant for association of ligand (L) and receptor (R); k_off_, rate constant for dissociation of complex LR; K_d_, equilibrium dissociation constant of reaction; [L], concentration of free ligand; [L]_o_, total concentration of ligand; [R], concentration of free receptors; [R]_o_, total concentration of receptors; and [LR] = [RL], concentration of ligand–receptor complexes. [L] = [L]_o_ − [LR]; [R] = [R]_o_ − [LR]. Equation (1) is the reaction scheme for the formation and dissociation of LR. Equations (4a) and (4b) are mass law equations.

In an ideal case, the drug after systemic injection exits from the vasculature and reaches the target such as location of the diseased cells, tissues or organs. To be successful, the amount of exiting nano drug must compete with its clearance from the blood. One way of successful competition is fast binding and internalization via a receptor located on the endothelial luminal surface. A favorable outcome depends on the number of available receptors, binding affinity, short residence time, and internalization rate. In an optimal situation, attached ligands (Figure 4a,b; L in Figure 5) recognize a plurality of receptors with moderate residence times (Receptors R in Figure 5). Multiple receptor-specific ligands are attached on each polymer platform to increase the number of permeating nano drugs per barrier. The affinities (indicated by values of K_d_^−1^) must be moderate to avoid receptor blocking by prolonged residence times (1/k_off_). In addition, long-lived high affinity antigen–antibody complexes such as TfR–aTfR (LR or RL in Figure 5) are prone to re-internalize and be sorted into the lysosomal pathway for degradation (Figure 4b) [19,52,53,54].

##### After Extravasation: Several Multiple Cellular and Extracellular Hurdles

After extravasation, the nano drug passes through several hindrances until reaching the site of its pharmacological activity. The hurdles besides vascular endothelial cell membranes are cell membranes (e.g., brain, breast and other organs), organellar membranes, and structural domains of intracellular organization and the extracellular matrix (ECM), such as amyloid-β aggregates in neurological disorders or abnormalities in tumor ECM. Membranes are compositionally and structurally variable depending on their localization and expression of specific receptors. Besides the dependence on energy required for molecule sorting and vesicle transport of nano carriers, the efficacy of transport through barriers may depend on ligand geometrical properties and affinity-guided selection to bind receptors [17,55,56].

Geometrical variables include size (diameter/length), shape (aspect ratio), flexibility (stiffness), and/or number of branches [22,57]. These properties can contribute to the physical fit of a nanoparticle passing a barrier [58,59].

##### Passive (Diffusive) Pathways

After intravenous (IV) injection of a nanoparticle, a primary effort is the exit from blood vessels through the endothelial barrier into interstitial tissue. Two major types of mechanisms are known: the diffusive pathway for small, nonpolar of hydrophilic molecules, and, in tumors, the enhanced permeability and retention (EPR) effect for the nanoparticle and the actively driven receptor-pathway extravasation [60,61]. In the case of a tumor, fast angiogenesis to the tumor can cause leaky blood vessels. These vessels of the growing tumor allow larger sized therapeutic agents, including nanoparticles or antibodies, to access the interstitial portion of the tumor to some extent.

##### “Active” Delivery Pathways

“Active” delivery involves ligands interacting with membrane receptors, which are part of a transport system assisting the opening of a barrier at the expense of energy [60]. One such barrier is the blood–brain barrier (BBB), which is a general term for the functionalities that organize the permeability of blood vessels in the central nervous system to precisely regulate the transfer of molecules between the blood and the brain [60]. The function of this barrier is to protect the brain from pathogens and neurotoxic molecules, as well as to maintain homeostasis. It allows permeation of molecules with Mw < 400 g/mol by passive diffusion [62,63], but it also allows larger molecules to enter such as transferrin or insulin, as well as nanoparticles that bind to the endothelial transferrin receptor (TfR), insulin receptor, or low-density lipoprotein receptor-related protein-1 (LRP-1) by the mechanism of receptor-dependent transcytosis [63,64,65,66,67,68,69,70,71,72,73,74]. While a normal BBB functions in healthy individuals, certain diseases can cause dysfunction of the BBB, as in the cases of brain tumor or Alzheimer’s disease [60].

##### Transcytosis Pathways, Vectors

The receptor-dependent extravasation from the blood involves specific binding to receptors at the luminal surface of the vascular endothelial layer, and then translocation along a specific route through the endothelial cell layer, followed by exit into the adjacent tumor interstitial tissue. The transcytosis pathways include receptor binding, receptor internalization, vesicle swapping via recognition of directional signals towards lysosomal, recycling, and the abluminal cell surface for exocytosis. 

The detailed mechanism of transcytosis is still elusive. A common feature is that it is led by “ferry” receptors, which do not dissociate from the transferred “vector” ligands on the way to the basolateral membrane. Persisting, high-affine ligand–receptor complexes were found to engage in the reverse reaction and are sorted into the lysosomal/degradation pathway [75,76].

##### Receptor-Driven Permeation through Cascades of Gated Barriers

**Figure 6:**
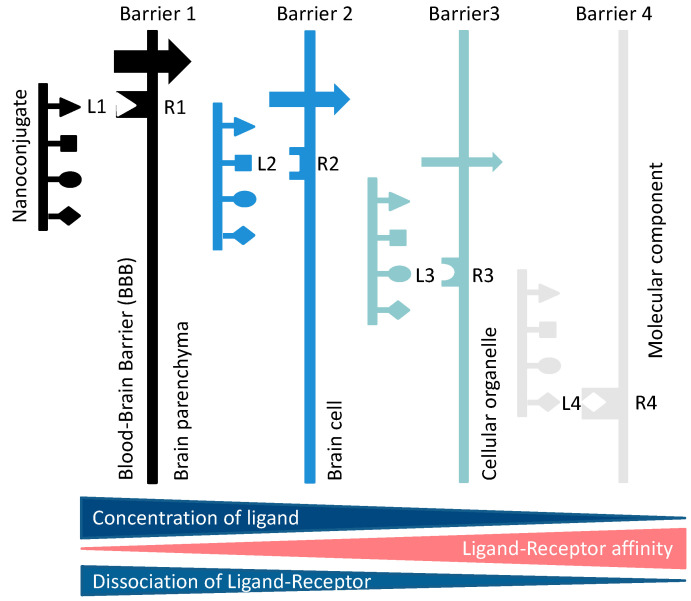
Cascade of reactions when a multi-ligand Li-containing nanoconjugate moves through a series of bio barriers with one specific receptor Ri at each structural border (i), such as membranes of brain cells or their organelles, to deliver a drug, e.g., ligand L4 on the nanoconjugate and R4 on the barrier’s effector site, to the number of barricades optionally. The movement is driven by the stepwise increase in affinities for binding ligands L1–4 to gating receptors R1–4. With an appropriate tuning of affinities and concentrations, an optimal deep flow through a given tissue can be achieved.

Examples of receptor-gated “transcytosis” pathways through the BBB are the TfR [77], insulin receptor [64], and lipoprotein (LDL-, LRP-1 receptor) pathways [33], among other pathways [62]. The routes can be used to access the brain parenchyma with a plethora of drugs attached to the specific receptor ligands. Such ligand peptides may be termed “shuttle peptides” or vectors. Delivery through the BBB is key for treatment of primary and metastatic brain tumors [61], as well as neurodegenerative disorders such as Alzheimer’s [78] and Parkinson’s diseases [61]; lastly, the BBB has to be overcome for deep tissue movement. The vectors of nano carriers are active in opening the BBB, and some, like the vector angiopeptide-2, specifically enter into brain cells or into parenchymal deposits (plaques) of peptides and other pathological tissue’s structure. Targeting a pathway through a multi-fence like border systems, called “cascade targeting”, involves specific carrier-bound molecules that bind to gating receptors specific for one of the barriers in the cascade. With the specific ligands on the nano carrier for one or several barriers in the cascade, the carrier can control access towards downstream-located targets within deeper regions of tissue. In the case of lacking a specific key ligand, the movement of the nano carrier might be stalled within an inactive compartment or carried by the target specificity of a nanocarrier co-loaded ligand within a non-cognate compartment. In another possibility, the key ligand could have a high affinity to an unintended receptor (e.g., of a key antibody to a receptor recognized as its antigen), and consequently being stalled in the antigen-residing compartment. Accumulation in the non-intended compartment could be prone to side effects and toxicity. Thus, the affinities between keys and receptors must be tuned. The pathway through a barrier cascade is illustrated in Figure 6. The case of receptor blocking at high ligand affinity is demonstrated in Figure 4 and Figure 5. 

##### Consideration of Size and Shape Effects on BBB Permeation

Deep into tissue movement during delivery from the bloodstream through vascular endothelium, interstitial tissue, ECM and cellular and intracellular membranes to the site of action has been shown to follow principles of receptor-targeted as well as random-passive permeation. Passive permeation (passive targeting) and targeted permeation (active targeting) are distinguished by the fact that receptor-targeted selection affords affine complex formation, energy-driven pathway sorting, and energy-driven membrane permeation. In both modalities, rates and amounts of delivered molecules are controlled by vehicle size, shape, rigidity, and surface properties. Surfaces could respond to hydrophilic, hydrophobic, electric charges, or acid–base sensitive groups.

In a passive case, the penetrating particle encounters extracellular and intracellular fluids with densities that vary with the concentration and nature of fluid molecules, semi-permeable membranes, and crosslinked peptide filaments as examples. The fluid molecules could be resting or streaming (bloodstream) in directions that are favorable/unfavorable depending on whether the particle and the environment move in phase. Moreover, particle size and shape may favor movements changing in different directions [22,34]. For example, spherical shapes of hydrodynamic diameter >10 nm hinder renal clearance, in contrast to EPR-mediated passive vascular extravasation, which is favored for particle spheres with diameters of 50 to 120 nm [57,61,79]. The chance to escape the blood flow by passive targeting is mostly unfavorable compared with receptor-capturing (active tumor targeting) [22]. Non-targeted nano carriers were designed for multiple cycles in the blood in order to increase the chance for escape. Sizes close to or below the renal threshold (4–8 nm) follow serum half-lives in the range of 0.5 to 1.5 h.

##### Transcytosis from Blood to Brain

Mini-nano-like drug carriers have been synthesized representing dendrimers, polymer nanoconjugates, and metal core particles in the absence of ligands [1]. However, the original sizes of a few nanometers may increase substantially when adding molecular layers and ligands for functionalization, and then the hydrodynamic size may extend into the >15 nm range, no longer qualifying as mini-nano carriers. Coiled polymers with hydrodynamic diameters <8 nm penetrate deeply into surrounding tissue within 1–3 h [17,22,34,80]. In one extreme, they can form compact coils that can be “wrapped” by membrane encounter before endocytosis and, in the other extreme, they can form an open version, presenting their ligands to receptors on the surface of membranes followed by internalization [17].

Receptor binding on the membrane surface of BBB initiates wrapping and uptake into travelling vesicles [21], and this may well require small ligand diameters. While details of the transcytosis pathway have not been fully elucidated, the vesicle–ligand–receptor entity moves through the cell under the energy-consuming control of specific proteins and encounters with other vesicles before exocytosis through the basolateral membrane, where the ligand dissociates from the receptor complex. 

## 2. Favorable Reasons to Use Mini Nano Vehicles for Delivery into Brain

### 2.1. Semiquantitative Description of Cross-BBB Delivery

#### Simplified Transcytosis Model

Delivery of nanomaterials to the brain is restricted by receptor-validated entry through the BBB. The nanocarrier is loaded with cargo (drugs) and vectors. The vector portion of the vector–cargo carrier recognizes the receptor of the transcytosis pathway through complex formation [1]. In the complex, the cargo remains covalently bound to the carrier. The complex with the receptor brings them together from the luminal side of the endothelial cell (i.e., the cell membrane surface adjacent to the bloodstream) to the abluminal side (i.e., the membrane surface at the opposite side, adjacent to the brain parenchyma), where the receptor–vector–carrier complex dissociates into cell surface bound receptor and the soluble vector–cargo–carrier ligand, releasing the vector–cargo–carrier into the parenchyma (Figure 4 and Figure 5). In the complex during transcytosis, receptor–vector is reversible binds by virtue of canonical structure. If the vector–receptor affinity is strong, a fraction of complexes may dissociate, yielding only a small portion of the overall amount of complex.

As summarized in Figure 4a, the ligand affinity to the receptor must allow easy dissociation from the transcytosis receptor to allow ligand forward permeation participating in downstream reactions or return to the blood vessel by a retrograde movement (Figure 4b). In the case of a high affinity ligand–vector–receptor complex, the complex could be trafficked into lysosomes and degraded [19,20,21]. Thus, treatment of a brain pathological conditions via a high affinity drug–vector–receptor complex would be less efficient owing to the limited carrier–drug amount than with a ligand having a moderate affinity and a higher amount of free carrier–drug. 

### 2.2. Calculation of Approximate BBB Permeation Efficacies Using Quasi Equilibrium and Other Approximations

The simplified schemes in Figure 4 and Figure 5 describe transcytosis as reversible complex formation of the ligand (i.e., vector-nanoconjugate) and receptor LR on the luminal side, and the dissociation of RL at the abluminal side of the vascular endothelial barrier, resulting in transportation of nanoconjugate and recycling of the membrane-bound receptor. While the receptor remains fixed to the cellular layer, the structures of LR and RL are assumed to be the same, with the vector–nanoconjugate–cargo ligand (mini-nano carrier) dissociating into the parenchyma. After dissociation, the vector–nanoconjugate may again encounter the receptor molecules, re-engage in complex formation, and return to the luminal side. For quantitative treatment, a *quasi*-closed system is assumed, which contains the membrane-bound free receptor, the receptor-complexed vector portion of the vector-conjugate, and soluble free vector–conjugate. In the simplifying assumption, the quantification considers only the dissociation–reassociation reaction of the receptor with the vector portion of the nanoconjugate at the abluminal membrane of the endothelial barrier, which is in equilibrium with the luminal side. Although the assumption appears superficial, it provides information about the amount of free receptor engage in complex formation, and thus influence the flux of the BBB permeating drug. The affinity is inversely correlated with the value of the dissociation constant K_d_ (Figure 5). The affinity is assigned to the vector as the active part of the nanoconjugate.

To calculate the concentration of the dissociated ligand, a *quasi*-equilibrium was assumed as an approximation (Equations (4a) and (4b) in Figure 5). After dissociation, the fraction of receptors that are ligand-free can cycle between the luminal and abluminal surfaces of the brain vascular endothelial barrier. As predicted by the mass law equations (Equations (4a) and (4b) in Figure 5), an increase in the ligand concentration ultimately results in the saturation of all receptor molecules, and thereby stalls transcytosis. In addition, the complexes with high affinity have prolonged residence times, 1/k_off_, that slow down transcytosis.

### 2.3. Effects on Transcytosis Efficacy at Selected Concentrations of Receptor and Ligand

The data presented in Table A1 were calculated for the conditions where [L]_o_ ≥ [R] (Equation (4a), Figure 5). Replacing “L” with “R” transforms Equation (4a) into Equation (4b) in Figure 5. The interpretation of the data after this transformation is different. For example, the relative transcytosis efficacy [R]/[R]_o_ in Table A1 transforms into [L]/[L]_o_, the ratio of the concentrations of free ligand ([L]) to total ligand ([L]_o_ = [L] + [LR], where [LR] is the concentration of the ligand-receptor complexes). For this new condition, [R]_o_ ≥ [L], the concentration of [R]_o_ must be tuned to [R]_o_ ≤ K_d_ in order to achieve optimal free ligand concentration after BBB crossing. 

High concentrations of free ligands on the abluminal side next to parenchyma are desired in cascade reactions (Figure 6), namely for binding and crossing of cellular membranes of neurons, astroglia, and microglia or binding to aggregates in the parenchyma. Commercialized affinity peptides identified by phage selection or similar methods mostly have receptor–peptide dissociation constants K_d_ in the range of 10 nM to 10,000 nM and function satisfactorily for vector-guided transport through biological barriers. Examples are listed above in Table 2.

### 2.4. The Dissociation Rate of the Ligand–Receptor Complex Is Coupled with the Affinity

In the section above on endothelial transcytosis, we considered the situation of limited pharmaceutical efficacy owing to a high ligand–receptor affinity. We have seen that a high affinity could favor cellular uptake, but that the invariably low degree of ligand–receptor complex dissociation can inhibit or even stall the efficacy of transcytosis. Furthermore, an unfavorable dissociation rate constant (k_off_ = K_d_ × k_on_) can decrease the flow of transcytosis, and thus kinetically inhibit the accumulation of free ligand on the abluminal side of the endothelial barrier. In addition, when the released ligand is consumed by competing reactions, the concentration of free ligand may be well below that needed for pharmaceutical efficacy.

#### 2.4.1. The Vector Part of the Ligands Matters

The importance of dissociation rates and binding affinities came into focus when antibodies against receptors were initially favored as optimal ferries through the endothelial layer of brain capillaries. In particular, these were antibodies against the TfR or insulin receptor. Dissociation constants of receptor–antibody complexes (K_d_) and rate constants of their formation and dissociation (k_on_ and k_off,_ respectively) had extreme values, with K_d_ = 0.1—5.0 nM, k_on_ = (10^4^ to 10^6^) M^−1^ s^−1^ and k_off_ = (10^−6^ to 10^−3^) s^−1^ [81,82,83,84,85,86], corresponding to residence times of hours and even days, which were unfavorable for drug delivery. In contrast, peptides with moderate receptor binding affinities of K_d_ = 0.1–5.0 µM, k_on_ = (10^5^ to 10^8^) M^−1^ s^−1^, and k_off_ = (10^−2^ to 600) s^−1^ [33,38,42,44,46,47,87,88,89,90] have residence times of 2 ms to 2 min, allowing a high degree of mass flow through the BBB.

#### 2.4.2. Polymalic Acid Tri-Leucine Group “Boosts” the Function of the Vector Group

In the example of the vector–polymalic acid trileucine nanoconjugate, the affinity of the transcytosis receptor complex of the peptide–vectors was “boosted” by coupling with the binding of the of the nanoconjugate to the BBB endothelial cell membrane [17] (Section 3.1). The finding sets an example for an affinity contribution by the vector microenvironment.

### 2.5. The Observed Impact of Vector–Receptor Affinity on Pharmaceutical Delivery 

Responsible for permeation efficacy are both ligand (vector)–receptor affinity (corresponding to 1/K_d_) and the ligand–receptor dissociation rate (k_off_ in Figure 5). Both affinity and dissociation rate are intrinsically connected parameters, and together add to the success of BBB permeation and pharmaceutical treatment. This remained historically unnoticed until it was found that the most affine IV-injected antibodies and conjugated drugs, as a rule, were unsuccessful as therapeutics. Under the pioneering work of W. M. Pardridge and coworkers [64,65,77], the concept of receptor-dependent transcytosis was originally introduced for drug delivery to the brain. They discovered that the antibody to the TfR could be used to “smuggle” molecules into the brain that were otherwise excluded from entering. The antibody was termed the “Trojan Horse”. Importantly, the discovery proved that the ligand adherence to the transcytosis receptor was conserved during the delivery. Their findings together with the affinity inferred limitation opened the door for providing efficient delivery to the brain for treatment.

#### The History of Drug Delivery to Brain

It was soon realized thereafter that the TfR, insulin, and other receptors such as the low-density LRP-1 [72] could deliver their natural or synthetic peptide vectors or protein-ligands into the brain via specific transcytosis pathways, and that recombinant fusions of polypeptides or biotin-streptavidin attached “cargo” ligands such as iduronidase [66], erythropoietin [67], or beta-secretase 1 (BACE1)-recognizing ligands could be delivered as well [68]. However, antibody-mediated transcytosis commonly has high antibody-receptor binding affinities (dissociation constants, K_d_, in the nanomolar and subnanomolar concentration range).

Limited dissociation of the TfR binding antibody was recognized as a problem and was soon resolved by replacing the wild-type TfR antibody with a low affinity recombinant [62,63]. Going one step further, Yu et al. discovered that they could enhance uptake and reactivity in brain parenchyma when one of the antibody binding sites had been engineered for binding (BACE1) [68,69], which is the peptidase that initiates the cleavage of amyloid precursor protein to amyloid-β in Alzheimer’s disease. As a consequence of the engineered substitution, the remaining original site had become less affine for binding TfR [69]. This indirect substitution effect on affinity resulted in the favorable dissociation. The finding was the impetus for employing low affinity receptor–peptide pathways, among them the low-density LRP-1 [72], binding the associating vector angiopep-2 (AP2) [32,33,70,71,72,73,74], as well as other pathways [37,42,45,46,47] (see Table 2). Receptor–affine peptide vectors (shuttle peptides [42,91]) are accessible by molecular display techniques or by the rational design of antigen–antibody mimicry peptides [23].

### 2.6. Transcytosis and Cascade Reactions

#### 2.6.1. How to Optimize the Flow through Cascade Barriers

Cascade reactions are considered here as one or several hurdles following the movement of a nanoconjugate through BBB. Inspired by the notion that the success of delivery deep into the brain could be limited by extreme affinities of nanoconjugate–vector receptor complexes, we were led to considering hurdles encountered by the nanoconjugate-ligand complexes. A possible way to maintain high flow would be to couple the cascade with a downstream decrease in dissociation constants, K_d(i+1)_ < Kd_i_, corresponding to the stepwise affinity increase illustrated in Figure 6. Ligand binding involves reversible binding to receptors R1, R2, and R3 and translocation through the cascade of barriers 1, 2, 3, and 4, whereby, after each barrier “i”, a higher ratio [L]/K_d(i+1)_ for still free ligand builds up in front of the next barrier “i + 1” and the receiving receptor is [R_i+1_] < [L]_o_ and [LR_i+1_] = [R_i+1_] [L]_o_/K_d(i+1)_ {1 + [[L]_o_/K_d(i+1)_}^−1^. For lim(K_d(i+1)_) → 0, [LR_i+1_] = [R_i+1_], i.e., the receptor at the terminal target side is engineered to be fully complexed with the ligand owing to its affinity. As discussed in previous sections, the condition for best flow, [L]/K_di,_ will be achieved if the K_di_ of each successive barrier–receptor is lower than the ratio for the preceding one. This requirement could be satisfied by selecting combinations of nanoconjugates containing peptides and/or antibodies with “tuned” K_d_ cascades. The given estimates are for simplified conditions. Not considered are reactions of complexes such as in- and outflow of ligands. In the case of drug delivery into brain, the intra brain cascade could begin with the binding to the transcytosis receptor at the endothelial/parenchyma membrane and then to a neuron cell membrane, membranes of organelles, nuclei, and so on, which are second, third, or fourth hurdles and their receptors, before the ligand finally arrives in the pharmacologically desired compartment and is consumed in a specific terminal reaction (Figure 6). Moving along the cascade in a strong mass flow towards the final receptor site is considered possible by a tuned stepwise increase in ligand–receptor affinities, thus favoring an “energy sink” at the end of the cascade. If the K_d_ values are tuned with concentrations of ligands, one-third of the nanoconjugate after passage, the concentration in the terminal barricade 4 would be reduced by a factor (3)^4^ = 81.

#### 2.6.2. Polymalic Acid Conjugates as Outstanding Candidates for Borderline Nanosized Drug Delivery Systems 

##### Structural, Chemical, and Physical Background for PMLA-Based Mini-Nanodrugs

The objective behind the design of mini-nanodrugs is to combine suitable features of both conventional and nano-sized pharmaceuticals. In order to achieve deep targeted delivery into tissue at a minimum of toxicity risk, in our opinion, the best approach is a design that favors (1) small sizes/high axial ratio, such a polymer as platform; (2) peptide targeted delivery; (3) simultaneous chemical attachment of a plurality of ligands (drugs, targeting groups, and imaging molecules); (4) designed hydrophilicity, hydrophobicity, and amphiphilicity; (5) the absence of bulky proteins; (6) biodegradability; (7) the absence of systemic toxicity; and (8) negligible immunogenicity.

##### Molecular Weight

As high-performance drugs, we developed mini-nanocarriers, mini-nanodrugs, and mini nano imaging agents that are conjugated along the linear structured polymeric PMLA platform with a molecular weight of 30 to 60 kg/mol (kDa). The conjugated platforms with various ligands have molecular weights that do not exceed 300 kg/mol (kDa). Examples of PMLA-based carriers and drugs are listed in Table 2. The small size and dynamic shape favor deep penetrations, attachment to vectors to bind arrays of receptors, and delivery of sufficient quantities of drugs. Controlled combinations with hydrophobic molecules can form amphiphilic segments for membrane fixation and subsequent specific binding to receptors for penetration, while locally dispersed ionic groups can favor, disfavor, and navigate the nanoconjugate’s spatial orientation optimizing approach and binding to charged membranes or macromolecules. 

##### The Linear Structure of the Polymeric Platform

PMLA of molecular weight 30,000 to 300,000 g/mol with the structure of a linear polyester (Figure 3) resembles a small molecule with a hydrodynamic diameter of 3.4–8 nm, a pH-dependent polyanion with pK_a_ 3.4, and a size-dependent zeta potential of −17 to −23 mV. The small, compact diameter in solution compared with the much more extended chemical structure is explained by an open to coil dynamic structure, which is enabled by the low energy rotation along the polymer backbone, and a short-range stiffness inferred by repulsion between negative-charged next-neighbor carboxylates. Rotation around the polymer axis can hypothetically result in an amphiphilic configuration with carboxylates on the hydrophilic side and methylene groups on the hydrophobic side. By conjugation of the many carboxylic groups with tri-leucine (LLL), the polymer is tailored for lipophilic response by the hydrophobic leucine-side chains and the shift towards neutralization of the pendant carboxylates under low acidic environment of pH ≤ 5 [92,93].

##### The Chemical Attachment of Ligands

The ligands of PMLA nanoconjugates are chemically attached to the pendant carboxylic groups of the polyester-forming malyl units [6,23,24,25,80,94]. Binding frequently includes linker molecules, which add distance between the polymer and the biologically functional group and may include a cleavage site to generate the active drug (Figure 3). Drugs attached by bifunctional PEG-linkers [17,23,80,95,96], which, in the case of prodrugs, can be cleaved at the pharmacological site of treatment (Figure 3) [25,95,97]. 

#### 2.6.3. Why Peptides Instead of Antibodies?

In principle, antibodies are disfavored because of their inherent size limitation for deep permeation and because of other disadvantages considered above. In mini nano constructs, they are replaced by peptides or other small receptor targeting groups with robust functions, preserving the minimum range of molecular weight sizes. In addition, peptides in cyclic configurations or mirror-imaged d-amino versions are the least biodegradable agents. LLL peptides are conjugated with 40% of the PMLA carboxyl groups, effecting high protection of the polyester against hydrolytic degradation [28,92,93].

## 3. Examples of Mini-Nano Devices

For an illustration of representative cases of PMLA-based mini-nano devices, four applications for mouse models are presented: (1) permeation through the BBB with the potential for imaging and cascade drug delivery in the treatment of tumor and neurodegenerative diseases, (2) high-intensity fluorescence imaging of tumor for guided resection of glioblastoma, (3) identification of tumors by MRI, and (4) the efficacy of nano drugs for inhibition of human HER-2 positive breast cancer. For a summary of mini-nano devices, see also Table 2. The well-established chemistry for activated PMLA-preconjugate and the substitution at the activated carboxylates with biologically relevant ligands are applicable [25].

The following examples demonstrate the overall competence of mini-nano devices in deep tissue delivery, in particular (1) providing access to brain parenchyma across the BBB, (2) tumor imaging, and (3) tumor treatment. Mini-nano devices have in common the polymer platform (Figure 3), ligands for receptor targeting, membrane binding and destabilization by attached LLL, PEG linker, and an optional fluorescent reporter dye, all assembled through covalent bonds. The abundant carboxylic groups provide anchorage for a plurality of drugs, targeting, and tumor imaging devices [17,25,34].

### 3.1. Example 1: PMLA-Based Mini-Nano Carriers (MNCs) for Delivery across the BBB 

Mini-nano carriers are macromolecules designed to penetrate into the brains of healthy mice and mouse models of Alzheimer’s disease and glioblastoma by transcytosis pathways of selected peptide vectors: angiopep-2 (AP2) vector of the LRP-1 pathway [72,73], cTfRL-peptide (human) [39] and B6-peptide (human, mouse) vectors of the TfR-pathway [33,40,41] and MiniAp-4 (M4, derived from bee venom) vector of a K/Ca ion-channel [42]. In the formula P(50 kDa)/LLL(40%)/peptide(2%)/rh(1%), P stands for poly(β-l-malic acid) (PMLA) and rh for rhodamine, whereas % refers to the fraction of malyl residues conjugated with the indicated ligand. MNCs containing Leu-Leu-Leu (LLL) are activated for enhanced (boosted) membrane permeation. The optical analysis of BBB permeation in normal brain, in brain of mouse models with Alzheimer’s disease, and in mouse brain tumors [98,99] and can give new insights into the intercommunications of different brain locations [100].

Mini-nano carriers of 50 kDa PMLA platform contain an average of 431 malyl-residues, 172 molecules of LLL, 8.6 molecules of vectors, and 4.3 molecules of rhodamine as the fluorescent reporter have hydrodynamic diameters <10 nm, zeta-potential of −2.2 to −16.5 mV, and molecular mass of 11.4 to 207 × 10^3^ g/mol [17].

The permeation across barriers is followed by ex vivo fluorescence microscopy at 534–558 nm excitation wavelength and 560–640 nm emission wavelength [17] of sliced tissue after IV injection and in vivo fluorescence labeled permeation of 14-week-old BALB/C and C57BL/6J (BL/6) mice (Charles River Laboratories, Wilmington, MA, USA) for normal brain BBB experiments [17], imaging and fluorescence guided resection experiments [34], and preclinical HER2-positive breast cancer treatment [23].

#### Kinetics and Efficacy of Mini-Nanocarriers’ Permeation through BBB

**Figure 7:**
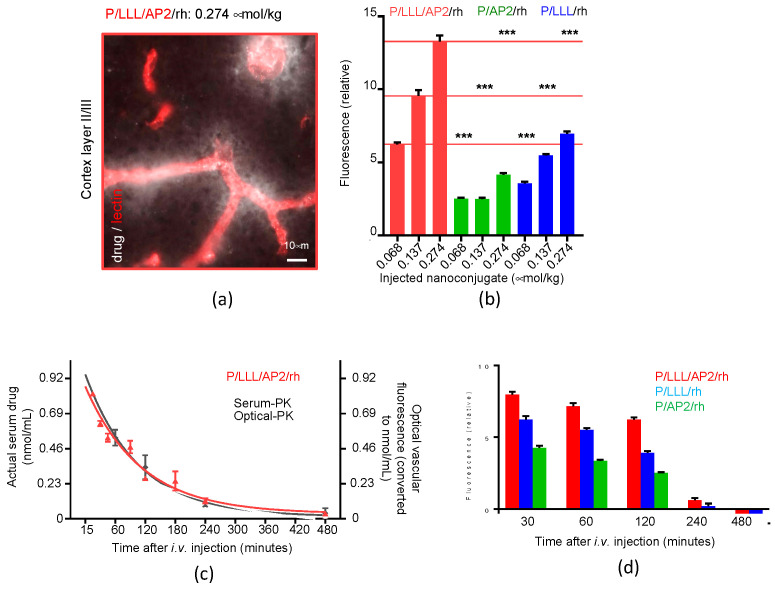
Mini nano carriers (MNCs) move across the blood–brain barrier (BBB) into brain parenchyma. (**a**) Ex vivo fluorescence microscopy of a mouse brain section from cortex layer II/III shows appearance of MNCs (P/LLL/AP2/rh) around the vasculature (in red) indicated as diffuse white material at 120 min after IV tail injection, converted to grey scale for quantification in the figure. (**b**). Quantitation of three mini nanoconjugates in brain sections (such as in (**a**)) is shown at different injected doses: P/LLL/AP2/rh (red), P/AP2/rh (green), and P/LLL/rh/ (blue). Comparison indicates the boosting effect of conjugated tri-leucine (LLL) on permeation of peptide vector Angiopep-2 (AP2). Significance level is *p* ≤ 0.0001 (***) with injected PBS as a reference. (**c**) Pharmacokinetics (PK) of P/LLL/AP2/rh in serum (black curve) compared with the fluorescence decay in the micro vessels of the brain and cortex layers II/III (red curve), after tail vein injection. (**d**) Early steady-state accumulation of P/LLL/AP2/rh is indicated in brain parenchyma between 60 and 240 min after injection, before major clearance from the vasculature (see (**c**)). Data plots and statistical analysis were conducted in Prism [17]. Reagent contents are as follows: P, poly(β-l-malic acid); rh, rhodamine and indicated vectors. Reproduced with permission from [17].

As shown in Figure 7, mini-nanocarriers containing PMLA, LLL, the vector AP2, and rhodamine (rh) are microscopically ex vivo detected for studying permeation of the brain capillary endothelial cell layer (BBB) of healthy mice (Figure 7a). Microscopical time- and region-dependent variations in fluorescence intensities of selected regions of interest were analyzed, which did not overlap with vessels and were corrected for lipofuscin autofluorescence (Figure 7b) [17,101].

The kinetics of the permeation BBB were indicated by fluorescence (Figure 7b) emerging from the brain capillary with the intensity depending on the type of vector, and were boosted by the presence of conjugated LLL. PMLA/rh in the absence of the peptides was permeation inactive. The optical method used in the experiment distinguished the permeating agents from lipofuscin of unknown composition in controls accumulated in neurons of aging normal mice and in large amounts in transgenic Alzheimer’s disease mouse models [17].

The distribution of mini nanoconjugates could be tracked deeply into parenchyma, but faded with time after 2 h and disappeared 4 h after injection using the time-dependent concentration in the blood system (pharmacokinetics) as a reference (Figure 7c). At fixed times, fluorescence levels were highest in the cortex and midbrain and lowest in the hippocampus, and correlated with the density of vasculature in these regions and increased with the dose of the injected mini nanoconjugates [15]. 

Pharmacokinetics were measured microscopically by following the decrease in vascular fluorescence intensity (Figure 7c, red curve). The kinetics in the parenchyma lagged behind the exponential fluorescence decay in the vasculature (Figure 7d), which reflected the influx from the capillary and a retrograde reflux to the blood vessels, in accordance with the bidirectionality of AP2 transcytosis through the vascular endothelium [70,71] and the absence of receptors that could have retained the reagent in the parenchyma.

### 3.2. Example 2: PMLA-Based Mini-Nano Imaging Agents (MNIAs) for Deep Brain Tumor Imaging by MRI Analysis and Near Infra-Red Fluorescence-Guided Tumor Resectinon 

#### Mini MRI-Contrast Agents

MRI in diagnosis of xenogeneic brain tumors has been performed using specific antibody-guided PMLA gadolinium imaging probes [80]. The probes had the general formula P/Gd-DOTA (10–12%)/mAb-tumor (0.12%)/MsTfR-mAb (0.12%)/Alexa-680 (1%). The MRI-enhancer was composed of PMLA polymer (denoted as P in the formula, with average Mw 74,000 g/mol), 1–2 molecules trastuzumab (anti-HER2 mAb) or 1–2 molecules cetuximab (anti-EGFR mAb), 1–2 molecules anti-mouse TfR mAb and 1–2 molecules anti-human TfR mAb, 62–74 molecules of gadolinium-tetraazacyclo dodecane tetra acetic acid (Gd-DOTA), and 6 molecules of Alexa Fluor 680 dye. 

**Figure 8:**
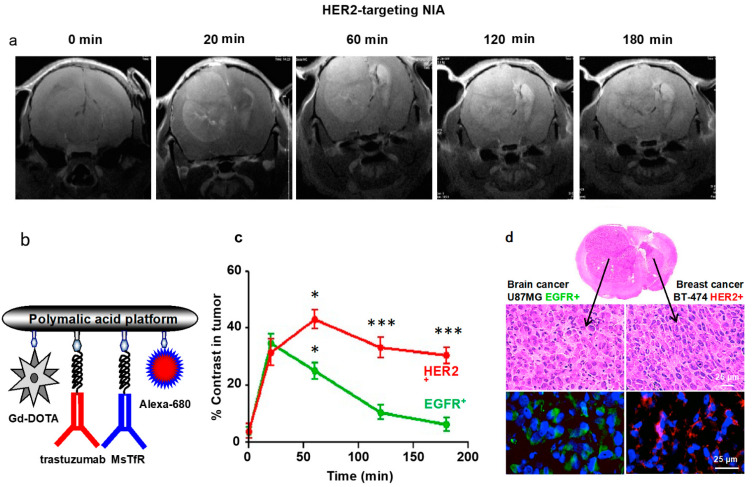
Tumor-specific nano MRI-contrast agents for virtual biopsy in the brain [80]. Example for specific detection of HER2^+^ metastatic breast tumor in the EGFR/HER2^+^ double-tumor nude mouse-model by analysis using HER2-specific nano imaging agent. (**a**) MRI scans of mice with double tumors, a primary GBM (U87MG, EGFR+) in the left hemisphere, and a metastatic breast cancer (BT-474, HER2^+^) in the right hemisphere. MRI at 20 min after IV injection of an agent specific for HER2^+^, (P/Gd-DOTA/ trastuzumab/MsTfR-mAb/Alexa-680, (**b**) together with an agent specific for the EGFR tumor. Contrasts were equal for both agents (**a**,**c**). Later in time, only the HER2+ tumor retained high contrast, while the contrast for EGFR faded (**c**). High contrast in the targeted HER2+ tumor was maintained at 3 h (* *p* < 0.05 at 60 min; *** *p* < 0.001 at 120 and 180 min). H&E-stained brain sections showed the presence of the two tumors (**d**) control by specific staining: HER2^+^ tumor (red), GBM (EGFR) (green). Gd-DOTA, gadolinium-tetraaza-cyclododecane tetraacetic acid; U87MG, glioblastoma multiforme; BT-474, HER2-positive breast cancer; TfR, transferrin receptor. Reproduced with permission from [80].

The probes had a hydrodynamic diameter of 16 nm and a zeta-potential of −7 to −9 mV [80]. The hydrodynamic diameters were larger, but in the range of mini-nano devices (17 nm probe, compared with <10 nm of MNDs), and functioned as contrast agents responding specifically to either EGFR or HER-overexpressing primary and metastatic human cancers in pre-clinical nude mice studies (Figure 8). The tumor-specific signal allowed localization of the tumors and their growth kinetics in the clinical diagnoses [80]. In follow-up experiments, the tumor-targeting antibodies were successfully replaced by angiopep-2 (AP2), a LRP-1 ligand for BBB transcytosis [35]. The new PMLA (Mw 60,000 g/mol)-based mini-contrast agent (MNIA) P/AP2(1%)/PEG600(GdDOTA)_3_(10%)/rh(0.5%) was synthesized following established chemistry [25,35,80], having a Mw of 270.3 g/mol, hydrodynamic diameter of 9.4 ± 1.6 nm, and zeta-potential of −8.2 ± 1.06 mV injected in 100 µmol Gd/Kg [35,80]. A further miniaturized probe of PMLA/PEG600(Gd-DOTA)_3_(10%)/AP2(1%)/rh(0.5%), PMLA(20,000 g/mol), and MNIA-Mw 89 g/mol, with a hydrodynamic diameter of 5.2 ± 1.1 nm, and a zeta-potential of −5.4 ± 0.41 mV, was, however, inactive.

### 3.3. Example 3: Image-Guided Resection of Glioblastoma 

**Figure 9:**
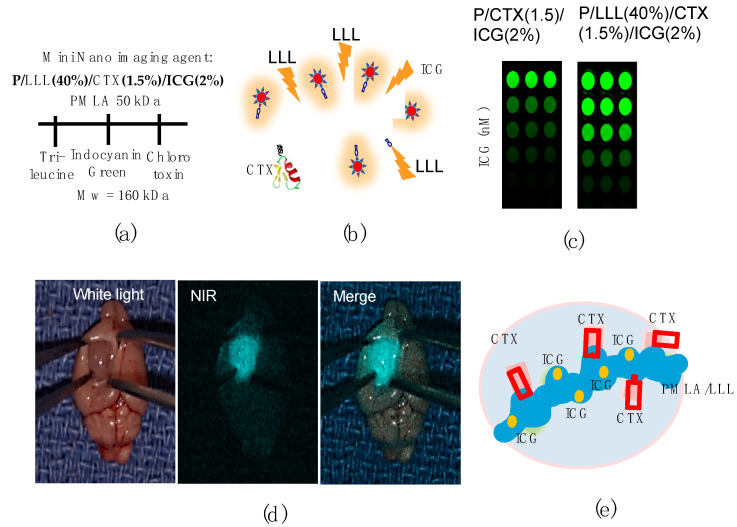
Imaging guided precision resection of glioblastoma. (**a**) Schematic of PMLA nanoconjugate as glioma-specific mini-nano imaging agent (MNIA) containing CTX for tumor targeting and ICG for near infrared (NIR) light emission stimulated by uptake into the tumor cells. (**b**) Schematic of nanoconjugate P/LLL(40%)/CTX(1.5%)/ICG(2%). Tri-leucine between conjugated ICG enhances fluorescence intensity [34]. (**c**) Strong fluorescence enhancement demonstrated by dilution comparing nanoconjugate with/out attached tri-leucine LLL. (**d**) Following fluorescence during resection using a hand-held detector. (**e**) Schematic model for P/LLL(40%)/CTX(1.5%)/ICG(2%) binding to tumor before internalization. (**a**,**d**) [34]. Reproduction from [34] with copyright permission of Elsevier.

Indocyanine green (ICG) fluorescence in conjugation with Chlorotoxin (CTX), a venom-derived peptide of the deathstalker scorpion (*Leiurus quinquestriatus*, molecular mass of 3996 g/mol), is a glioma targeting device when conjugated with PMLA/LLL [34] (Figure 9 and Table 2). CTX has affine targeting ability for glioblastoma, and ICG is an FDA-approved NIR-fluorescent agent [34] with the composition P/LLL(40%)/CTX(1.5%)/ICG(2%) (Figure 9), with NIR fluorescence at 800 nm wavelength (570/600 nm excitation). The IV injected mini-nano agent accumulates in the brain [43,44,45]. Tri-leucine peptide LLL, when co-loaded to the agent, induces a sevenfold increase in fluorescence intensity (Figure 9a–c).

The specificity of the MNIA agent P/LLL/CTX/ICG was studied by flow cytometry and fluorescence microscopy [34], comprising binding to glioma cells and competition with constituents of the NMIA. Distinct and overlapping binding sites are identified for CTX and PMLA/LLL. The attachment of P/LLL(40%) to cell membrane is supported by energy-based structure calculation [34,92,93]. Cooperativity of PMLA/LLL- and CTX-binding involves structural rearrangement of the membrane for permeation [17].

### 3.4. Example 4: PMLA-Based Mini-Nano Drugs for the Treatment of HER-Positive Breast Cancer 

#### Conventional Cancer Targeting by Antibodies

**Figure 10:**
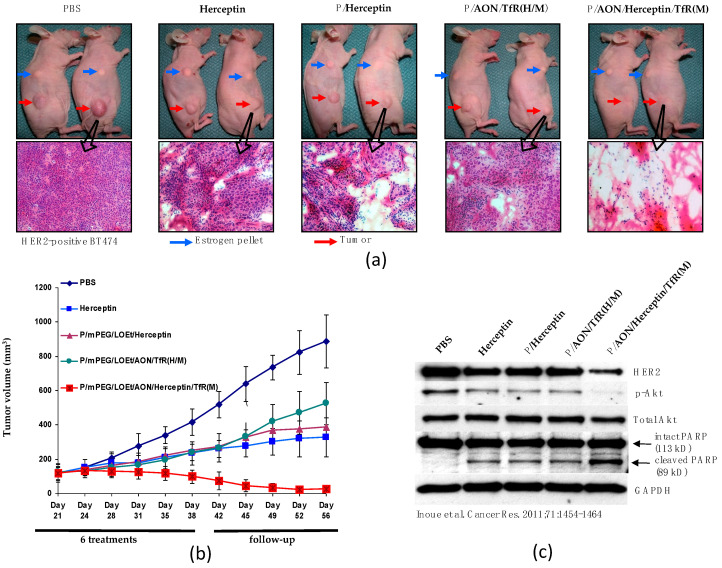
Growth inhibition of human HER2^+^ breast tumor in preclinical studies of nude mice treated with the conventional PMLA-nanoconjugate containing HER2-antisense oligonucleotide (AON_Her2_ blocking mRNA_Her2_), trastuzumab (Herceptin), and anti-mouse TfR antibody (m-TfR). Lead nanodrug P(100 kDa)/mPEG(5%)/LOEt(40%)/AON/Herceptin/m-TfR) [94]. (**a**) Mice after controls or multiple injection of the nanodrug or synthesis intermediates displayed in inset (**b**); after multiple injections in upper images (**a**). H&E staining in lower images (**a**). (**b**) Inhibition of tumor growth over time. (**c**) Western blotting after injection of PBS control, lead nano drug, and synthetic intermediates. Akt, serine/threonine-specific protein kinase; GAPDH, glyceraldehyde-3-phosphate dehydrogenase; HER2, human epidermal growth factor receptor 2; LOEt, leucine ester; PARP, poly(ADP-ribose) polymerase; PBS, phosphate-buffered saline; PEG, polyethylene glycol; P or PMLA, poly (β-l-malic acid). Reproduced under AACR Author reuse license from [94].

Pharmaceutically nano drugs based on PMLA have been proven to be successful for treating human cancers carried in mouse models [6,14,24,25,80,96,97]. An example is the treatment of HER2+ (positive) breast cancer with the nano drug P(100 kDa)/mPEG(5%)/LOEt(40%)/AON/Herceptin/m-TfR, which resulted in the complete regression of the breast cancer [24] (Figure 10). This treatment required escape of the nano drug from the vasculature and subsequent permeation of the tumor matrix and tumor cell membranes achieved by the combined sequential action of antibodies against TfR and anti-HER2 (Herceptin). Because the synthetic nanodrug contained the two targeting antibodies, it had a hydrodynamic diameter of 22.1 ± 2.3 nm and a zeta-potential of −5.2 ± 0.4 mV, thus not qualifying as a mini-nanodrug (<10 nm). The nanoconjugate contained l-leucine ethyl-ester (LOEt(40%)) for permeation through endosomal membrane into cytoplasmic in order to achieve AON delivery for the inhibition of HER2 synthesis [24].

**Figure 11:**
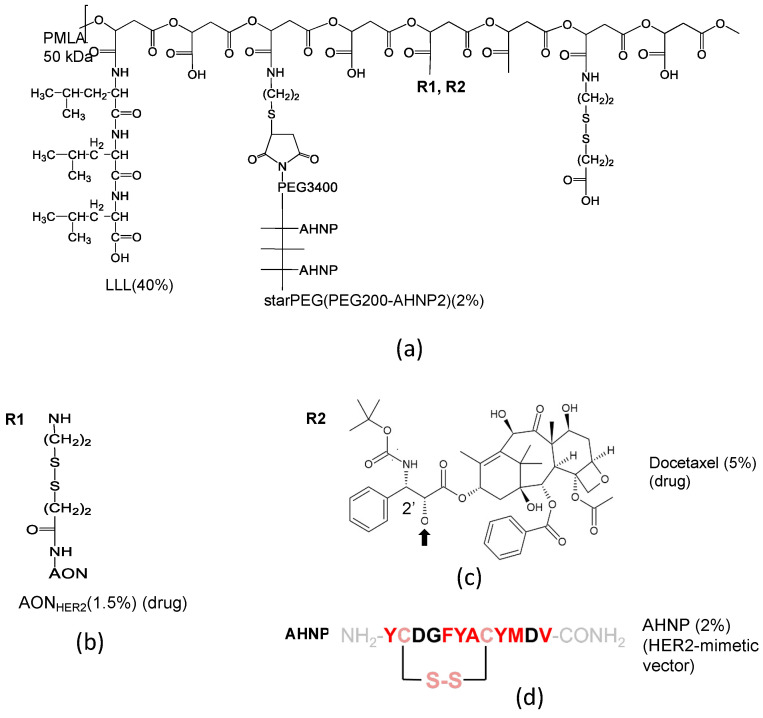
Synthesis of mini-nano drugs replacing antibodies by HER2 affine peptide. (**a**) Structure of the MNDs. (**b**) AON_HER2_, antisense oligonucleotide (AON) used in experiments in Figure 11 and Figure 12. (**c**) Inhibition by Docetaxel used instead of AON. (**d**) HER2-mimetic vector (anti-HER2/neu-peptide AHNP (hydrophobic amino acids in red); HER2, human epidermal growth factor receptor 2; LLL, tri-leucine; PEG, polyethylene glycol; PMLA, poly(β-l-malic acid). Reproduction with permission [23,25].

**Figure 12:**
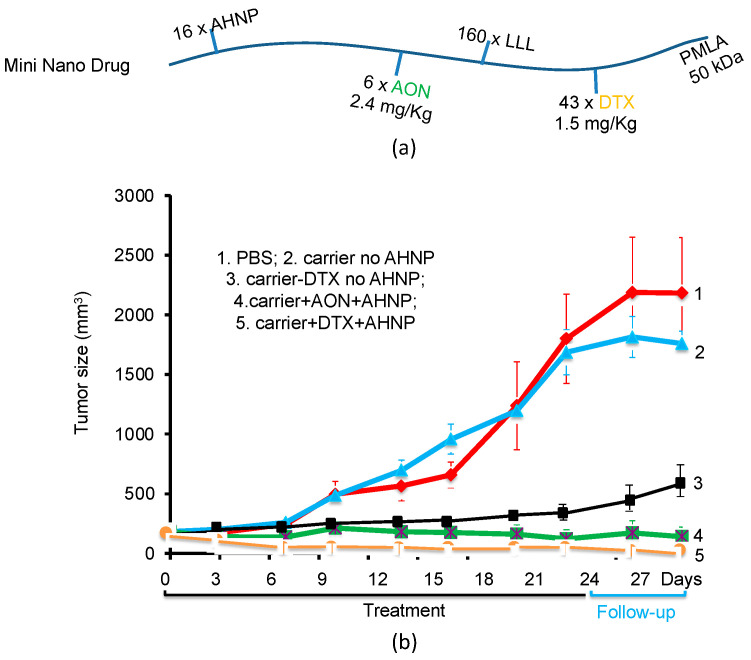
Mini-nanodrugs that inhibit growth of HER2-positive breast cancer. (**a**) Schematic drawing of mini-nanodrugs indicating numbers of ligands: AON_HER2_ (6 molecules) [23] or docetaxel (DTX, 43 molecules). (**b**) Growth inhibition following injections of mini-nanodrug containing AON, DTX, and LLL. AON, antisense oligonucleotide; AHNP, anti-HER2/neu peptide; HER2, human epidermal growth factor receptor 2; LLL, tri-leucine; PBS, phosphate-buffered saline; PMLA, poly(β-l-malic acid). Reproduced and modified under Creative Commons License from [23].

By replacing the antibodies with the HER2-mimetic peptide, AHNP (Figure 11 and Figure 12) and the LOEt with the non-toxic tri-leucine LLL, the mini-nanodrug [23] was synthesized. To prevent the designed peptide AHNP from refolding onto P/LLL, it was conjugated with an eight-arm linker starPEG [23]. The mini-nano carrier with the composition P(50 kDa)/LLL(40%)/starPEG(PEG200AHNP_2_)(2%)/AON(1.5%) [23] was ligated with either AON, 5′-CATGGTGCTCACTGCGGCTCCGGC-NH_2_-3′ for inhibiting mRNA-directed HER2 synthesis [25], or with toxic docetaxel (DTX, 5%) (Figure 11c). In Figure 11, DTX was esterified with PMLA via the acid-labile link in the 2′-position of DTX [102]. The mini-nanodrug P(95 kDa)/LLL(40%)/starPEG(PEG200-AHNP)_2_(2%)/DTX(5%) (Figure 11) The mini-nanodrugs had a hydrodynamic diameter of 7.8 nm. After the PMLA(LLL40%)-induced membranolytic release into the cytoplasm [92,93] of the HER2+ cancer cells, significant growth inhibition was achieved (Figure 12), which was comparable with the treatment by the antibody-containing nanodrugs [94].

## 4. Summary of Distinguished Features of PMLA-Based Mini-Nano Devices 

### 4.1. Multifunctionality of the Mini-Nano Device

Presented above are several cases of mini-nano devices that function as vehicles across normal and tumor (BBB) barriers performing high-grade delivery, imaging, and antitumor treatment. The signature structure of mini-nano devices contains PMLA and other similar structured polyfunctional polymers as single platforms. The single bonded polymer backbone displays a high degree of conformational freedom, allowing the display of multiple attached ligands and chemically reactive groups. In response, the platform is able to adopt a variety of linear and coiled conformations of low energy receptor-specific interactions. The moderate PMLA UV intrinsic absorbance below 250 nm wavelength and the moderate zeta-potential of free and ligand-substituted PMLA allows a short distance to macromolecules and membranes. Biodegradability, either spontaneous or enzymatical, excludes long-lasting depositions, minimizing immunogenic and toxic side effects [14]. The relative short PK-t_1/2_ in blood and yet a sufficiently effective escape into vascular endothelial layers render mini-nano devices valuable tools for imaging and treatment purposes, especially of CNS.

### 4.2. Cascade Targeting Affording Attachment of Several Peptides per MNC 

The simple chemical attachment of multiple functional groups at the polymer platform opens the door for efficient delivery downstream of multiple barriers (Figure 6). 3D-printed Transwell [103] or spheroid [104] models offering precise cells layers could be used in the development of such cascade routes. Optical tracing can provide information about a time-resolved microscopic location of the fluorescent MNC under the influence of the microenvironment, barrier receptor affinity, size, length, and shape [17]. Brain barrier, permeation through the BBB, the extracellular matrix, basal lamina, pericytes, astrocytes, and neurons could be studied. In addition, retrograde reactions can be discovered by comparing MNC extra vascularization kinetics, residing times, and serum (PK) clearance kinetics [17].

### 4.3. Optimal Settings of Mini Nano Devices (MNDs)

There are several possibilities for improving the efficacy of MNDs: (1) An appropriate choice of reversibly bound ligands that have ligand–receptor dissociation constants K_d_ > 10 nmol/L to avoid carriage blocking. (2) Increase the percent loading with receptor ligands, but being careful to not overload owing to molecular crowding that could reduce efficacy [15]. (3) Load the nano conjugate platform with a mix of shuttle peptides (vectors) targeting several routes of cross-BBB-pathways, but again being aware of molecular crowding. (4) Allow an increased uptake-time into the brain vascular endothelial cell by prolonging blood circulation. Achieve this by attaching stealth mPEG5000 (2–5%) onto the nano conjugate. Be aware that this measure can lead to deposition and cytotoxicity. (5) Principal possibility of selective or deep movement into tissue by the use of barrier-specific receptor ligands (see cascades); however, technology is not well developed. Use a vector in the terminal cascade-position with highest possible affinity or replace with an irreversible terminal reaction (Figure 6).

## 5. Comparison with Non-PMLA Types of Mini-Nano Devices

Gadolinium benzyl diethyl triamine penta-acetic acid (Gd-Bz-DTPA) functionalized poly amido-amine (PAMAM) dendrimers with diameters of 11.7 to 11.9 nm have been applied for glioblastoma MRI [54]. HPMA copolymer-based conjugates for the delivery and controlled release of retinoids with hydrodynamic diameters of 7.4 nm to 12 nm have been synthesized and reported to induce the differentiation of retinoid-responsive HL-60 cells [105]. Other MNCs named ultra-small nanoparticles (USNPs) are reported that contain diverse, mostly inorganic core particles, in particular gold (USAuNPs) and iron oxides [60,106]. However, small-sized spherical metal particles such USAuNP below 10 nm in diameter can strongly deviate from matrix-imbedded metal particles of >15 nm in affinity, stability, toxicity, and biodistribution [10,106]. Aggregated, USAuNPs and other aggregated metal particles have significantly higher affinities and longer circulation times, and clear from the blood through the liver. Free metal surfaces can cause toxicity by radical formation, reactive oxygen species (ROS) production, and aggregation [107,108,109,110]. Historically, quantum dots consisting of a variety of inorganic cores have been introduced because of their attractive intensive light emission; however, they can be toxic owing to the exposure of their metal cores. Light-emitting “conjugated polymer nanoparticles” (CPNs) on organic chemistry basis have been developed, which contain conjugated cyclic carbon systems for imaging and theragnostic purposes [111], and compete with the application of inorganic quantum dots. Mini-nano PMLA-platforms are organic and biodegradable by spontaneous hydrolytic cleavage or by hydrolytic enzymes into reusable building blocks or water and CO_2_. Although the organic ultra-small quantum dots are comparable to PMLA mini nano imaging (MNIA) reagents in size, they are different in the chemistry of their not nature-derived platforms, containing branched cyclic or metal core components, owing to toxicity, not undergoing reversible helix-coil structural changes (involving low to high axial ratios owned by PMLA-built agents), not undergoing degradation to ultimately water and carbon dioxide, yet forming radicals or long-lived depositions of immune responsive and toxic material. Nevertheless, their application still lies in the field of imaging with superior resolution [1].

The new developments are organic ultra-small quantum dots, which are comparable in size to PMLA-derived mini-nano imaging agents. However, their different biochemistry including the possibility of toxicity is unsolved. Nevertheless, their application still lies in the field of imaging with superior resolution. 

## 6. Summary and Conclusions

Small-sized co-polymers are excellent candidates for targeted deep tissue drug delivery and imaging over relatively short exposure times and when nature-derived, providing a high degree of safety. Unlike USNPs (i.e., core metal nanoconjugates), mini nano devices (MNDs), containing a choice of natural derived and organic chemistry and owing to their molecular size compared with conventional pharmaceutics, may combine proven advantageous drug properties. Their tissue accessibility in general rests on their hydrodynamic shape and small size and, in particular, on the amphiphilic structure of polymalic acid nanoplatform in the favorable chosen example. While their general size and shapes favor a broad tissue distribution, their tissue specificity can be modulated by their multiple cargo in combination with the amphiphilicity of the platform. This multiplicity renders them highly variable in the choice of tissue targetability, biocompatibility, and tissue penetration depth. Peptides by means of ligands with variable low and high receptor affinities are selectable as ligands for high and low receptor affinity without dramatically increasing the size of the nanodrug. The multivalent mini nanoconjugates of PMLA are structurally and functionally programmed by the attachment of a variety of small molecules without giving up their qualification as mini-nano devices. Nevertheless, antibodies or other large molecules can be co-attached for inferring prolonged blood circulation or specific immunological activities, but at the same, the substitutions are prone to negatively affecting the original advantages of the small-sized carriers. In another aspect, although metal core USNPs are powerful small nanodevices for imaging, their stiff physical shape may exclude combined contacts of several ligands with surface-distributed receptors on cell targets. Mini-nano carriers by their linear arrangement of loaded ligands and the dynamic adaptability of the polymeric platform could combine interactions with difficult to approach cell surfaces. The examples described in Section 3 elucidate the functions of mini nano devices in image-guided tumor diagnosis; image-guided resection and drug treatment; and, in all cases, a deep permeation of targets. Because of the numerous applications, PMLA mini-nano carriers have the potential [112] to move to the pharma market after upscaling their production chemistry. Importantly, their dynamic structure and multiple small-sized cargo ligands favor simultaneous receptor binding for theranostic and combination therapy. The versatility significantly challenges new ideas for application in clinics.

## Figures and Tables

**Table 1:** Design of a hybrid construct representing a classical chemotherapeutic and minimized nanodrug.

Feature	Verification	Effect
Size (volume)	Low mass, hydrodynamic diameter < 10 nm	Fast and deep penetration
Shape	High axial (high aspect) ratio	Fast and deep penetration assisted by geometry
Platform molecularity	Single molecule	Stability against spontaneous disassembly
Functionality	Multiple ligands	Multiple targeting and delivery
Ligand attachment	Covalent	Controlled assembly
Drug attachment	Reversible if prodrug	Controlled release
Targeting	Multiple targeting through gated bio barriers	Tuned affinity-gating receptors regulate movement through cascades
High affinity targeting Low affinity targeting	Affinity locked ligand-receptor Speedy delivery via ligand-receptor	Lock-in for antigen-antibody capture Transport through multiple junctions

**Table 2:** Examples of mini-nano devices including mini-nano carriers (MNCs), mini-nano imaging (MNIAs) agents, mini-nano drugs (MNDs), and their attached peptide vectors.

Mini-Nano Device (MNC, MNIA, MND) and Peptide Vector	Formula ^a^	MW (g/mol)	Size ^b^ (nm)	ζ-Potential (mV)	K_diss_^c^ (μM)	Dose ^d^ (µmol/kg)	Serum t_½_ (h) ^e^	Site t_½_ (h) ^f^
Platform	poly (β-l-malic acid) (PMLA)	50 × 10^3^	3.3 ± 1.5	−16 ± 0.9	-	-	-	-
*Angiopep-2 (AP2) Vector*	TFFYGGSRGKRNNFKTEEYC [32,33]	2404	-	-	0.33 [32,33]	12–30 [32,33]	-	-
MNC [34]	P(50 kDa)/LLL(40%) /PEG3400-AP2(2%) /rh(1%) [17]	165 × 10^3^	4.5 ± 1.5	−11.6 ± 1.8	-	0.068–0.548 [17]	1.2 [17]	2–3 [17]
MNIA (MRI contrast)	P(60 kDa)/PEG600(Gd-DOTA)_3_(10%)/AP2(1%) /rh(0.5%) [35]	270 × 10^3^	9.4 ± 1.6	−8.2 ± 1.72	-	-	-	-
*Fe-mimetic Vector: cTfRL*	CRTIGPSVC (S-S disulfide bridge) [36]	932	-	-	-	5–40 [37,38]	-	-
MNC [17]	P(50 kDa)/LLL(40%) /PEG2000cTfRL(2%)/rh(1%) [17]	142 × 10^3^	-	−9.58 ± 1.1	-	0.068–0.548 [17]	-	-
*TfR-mimetic Vector: B6*	CGHKAKGPRK [39,40,41]	-	-	-	-	-	-	-
MNC [17]	P(50 kDa/LLL(40%) /PEG2000B6(2%)/rh (1%) [17]	153 × 10^3^	-	−6.1	-	0.068–0.548 [17]	-	-
*MiniAp-4 Vector* *M4*	H-[Dap]KAPETALD-NH_2_ (Dap-d lactam bridge)	911	-	−10.4 ± 1.3	-	0.2–1.04 [42]	-	-
MNC [17]	P(50 kDa)/LLL(40%) /PEG2000-M4(2%)/rh (1%) [15]	139 × 10^3^	-	-	-	0.068–0.548 [17]	-	-
*Chlorotoxin Vector (to glioma)* CTX	MCMPCFTTDHQMARKCDDCCGGKGRGKCYGPQCLCR; (CTX) [43,44,45]	3996	-	-	0.66 [23]	0.05–0.15 [44]	-	-
MNIA (fluorescence)	P(60 kDa)/LLL(40%) /PEG2000-CTX(1.5%) /ICG(2%) [34]	160 × 10^3^	11.8 ± 16	−20.5 ± 1.8	-	-	1.5 [34]	9.5 [34]
*Vector to HER2* (HER2-mimetic) [23] AHNP	YCDGFYACYMDV-NH_2_ (S-S disulfide bridge)	1647	-	-	0.52 [23,46,47]	-	-	-
MND [23]	P(50 kDa)/LLL(40%) /StarPEG(PEG200 (AHNP)_2_(2%) /AON(1.5%)	331 × 10^3^	7.8 ± 2.1	−13.8 ± 1.3	4.6 [23]	0.75	-	10
MND	P(50 kDa)/LLL(40%) /StarPEG(PEG200 (AHNP)_2_(2%) /DTX(5%)	274 × 10^3^	-	-	-	5.0	-	-

^a^ % fraction of PMLA malyl residues conjugated with ligand at their free –COOH. ^b^ Molecular mass calculated according to formula. ^c^ Dissociation constant of mini nanoconjugate complexed with host receptor. ^d^ Dose per mouse, concentration range of conjugated vector residues in experiments. ^e^ Serum half-life. ^f^ Half-life of mini nanoconjugate at targeted site. AHNP, anti-HER2/neu peptide; AON, antisense oligonucleotide; DTX, docetaxel; Gd-DOTA, gadolinium-tetraaza cyclododecane tetra acetic acid; ICG, indocyanine green; LLL, tri-leucine; P, poly(β-l-malic acid); PEG polyethylene glycol; rh, rhodamine; cTfRL, transferrin receptor ligand.

## Data Availability

No experimental data reported.

## Appendix A

### Model Calculations

The distribution in free ligands or receptors exemplified by the quasi-equilibrium approach. The calculations in Table A1 are based on Equations (4a) and (4b) in the reaction scheme in Figure 5. We restricted the conditions so that [L]_o_ ≥ [R]_o_ in Equation (4a) and [R]_o_ ≥ [L]_o_ in Equation (4b), implying that the concentration of the ligand–receptor complex [LR] is negligible against concentrations [L]_o_ and [R]_o_, as indicated. In the following representative cases #5, #6, #11, and #17 (Table A1) with concentrations [L]_o_ > [R]_o_ and [R]_o_ < K_d_ = [L]_o_, we find that the relative activities [R]/[R]_o_ are highly favorable (50%). In cases #7 and #8 (similar conditions to cases #5 and #6, except for increased [L]_o_), the relative activities are 33% and similar to case #10, with higher values of K_d_, [L]_o_, and [R]_o_. Cases #12 to #16 all show excellent relative activities (range of 83% to 95%) at further increased values of K_d_ > [L]_o_, [R]_o_. In contrast, when the relative differences between K_d_ and [R]_o_ were reduced, as in cases #1, #2, #3, #4, and #9, the relative activities became highly unfavorable (<10%). The examples illustrate that the concentrations of free ligand and receptor must be lower than the value of K_d_ in order to achieve high [R]/[R]_o_ relative receptor ratios, i.e., permeation efficacies. It should not be overlooked that this favorable ratio [R]/[R]_o_ is restricted by the condition [L]_o_ ≤ K_d_. Thus, high transcytosis is achievable for low vector–receptor binding affinities. Otherwise, transcytosis is not efficient or even stalled at the highest concentrations of [L]_o_ > K_d_ ≤ 1 nM.

**Table A1:** Transcytosis efficacy is expressed as the ratio of the concentration of free receptor to total receptor ([R]/[R]_o_).

Case #	K_d_ (nM)	[R]_o_ (nM)	[L]_o_ (nM)	[LR] (nM)	[R] (nM)	[R]/[R]_o_ (%)
1	1	1	10	0.909	0.091	9.0
2	1	2	10	1.818	0.182	9.1
3	1	1	20	0.952	0.048	4.8
4	1	2	20	1.905	0.095	4.8
5	10	1	10	0.5	0.5	50
6	10	2	10	1.0	1.0	50
7	10	1	20	0.667	0.333	33
8	10	2	20	1.333	0.667	33
9	10	4	100	3.696	0.364	9.1
10	50	4	100	2.667	1.333	33
11	100	4	100	2.0	2.0	50
12	100	1	10	0.091	0.909	91

For K_d_ ≥ [L]_o_ > [R]_o_ and values of K_d_ = 1 nM to 10 nM, concentrations of receptor = 1–10 nM and concentrations of vector = 10–1000 nM. Data were calculated using Equation (4a) (see Figure 5). K_d_, equilibrium dissociation constant of vector–receptor complex formation; L, vector portion of the nanoconjugate; [L]_o_, total concentration of vector contained in the nanoconjugate (L); R, receptor binding the vector; [R], concentration of free receptor; [R]_o_, total concentration of receptor; [LR], concentration of ligand–receptor complexes.
